# Submicron quantum dot light-emitting diodes enabled by pixelated topological meta-mirror

**DOI:** 10.1515/nanoph-2024-0543

**Published:** 2025-01-08

**Authors:** Taikang Ye, Dadi Tian, Dan Wu, Xiao Wei Sun, Kai Wang

**Affiliations:** State Key Laboratory of Optical Fiber and Cable Manufacture Technology, Institute of Nanoscience and Applications, and Department of Electronic and Electrical Engineering, 255310 Southern University of Science and Technology, Shenzhen 518055, China; Department of Electrical and Computer Engineering, National University of Singapore, Singapore 117583, Singapore; Pengcheng Laboratory, Shenzhen 518055, China; College of New Materials and New Energies, Shenzhen Technology University, Shenzhen 518118, China

**Keywords:** micro-QLEDs, topological metasurface, meta-cavity

## Abstract

As a highly competitive display technology, the realization of pixelated full color quantum dot light emitting diodes (QLEDs) is an indispensable step for high resolution display. Meanwhile, with the rise of near eye display, a submicron pixel size is required for a high-resolution display within a small area less than 1 inch. However, the realization of submicron full color quantum dot pixels by direct patterning is still a big challenge. In this work, we propose a topological meta-mirror structure for the realization of submicron RGB QLEDs. The pixelated topological meta-mirror is introduced with a sufficient design freedom. A powerful light manipulation capability is offered by the topological meta-mirror even with limited period number, which enables the construction of RGB meta-cavities. The pure RGB emissions from meta-cavities can be realized with energy ratios larger than 88 % based on optimized topological meta-mirrors. For a subpixel size of 1 μm, the energy ratios for target color emission can still be larger than 85 %, which indicates a pure color emission. And a minimum subpixel size of 0.6 μm and an ultra-high pixel density of 21,666 pixel per inch can be realized with a 3 × 3 topological meta-mirror array. The proposed meta-cavity structure based on topological meta-mirror provides a new technique route for full color QLEDs especially for high pixel density required scenarios.

## 1 Introduction

Based on the excellent luminance property [1], [2], [3] of colloidal quantum dots (QDs) such as narrow emission spectrum, tunable emission peaks, and high luminescence efficiency, quantum dot light-emitting diodes (QLEDs) have attracted many attentions. During the past decade, remarkable progress in QLEDs performance improvement has been reported [4], [5], [6], [7], which also demonstrate its promising application in the emerging display technology [2], [5], [8], [9], [10], [11]. The rise of near eye display applications such as virtual reality (VR) and augmented reality (AR) requires a high-quality display on a small screen with an effective display area less than 1 inch, which sets an ultrahigh standard in pixel density for next generation display technology [12], [13], [14]. For example, to realize a full color (RGB) 4K resolution (3,840 × 2,160 × 3) display within a 1 inch (diagonal) screen, an ultra-high pixel density larger than 13,000 pixel per inch (PPI) is required, which is corresponding with a subpixel size less than 2 μm or even submicron size for a smaller screen size. However, the high pixel density patterning with submicron subpixel size is still a critical barrier for full color micro-QLEDs.

Due to the colloidal state of RGB QDs in solution, the precise patterning of QDs cannot be realized by vacuum deposition technology, which has been widely used for micro-OLED fabrication. Currently, the realization of full color micro-QLEDs array is mainly achieved by inkjet printing [15], [16], [17], [18], [19], [20], transfer printing [21], [22], [23], photolithography [24], [25], [26], [27], and electrophoretic deposition [28], which require a precisely patterning of QDs layer. However, the direct patterning of QDs layer still faces many challenges such as uniformity and roughness control, especially for a high-resolution pixelated array consisting of millions of QDs subpixels. Meanwhile, complex driven circuits for separate RGB QLEDs based subpixels further increase the cost of full color QLEDs in practical applications. To conquer this problem, optical cavity based full color patterning methods [29], [30] are reported to realize full color emission without directly patterning of QDs layer. By introducing a patterned phase modulation layer, the Fabry–Perot (FP) cavities with different thickness can realize a full color emission. The problems faced by directly patterning of QDs layer can be avoided, and the pixel size is determined by the patterned phase modulation layer. However, due to the different cavity thickness requirements for different color emissions, multiple times photolithography and alignment are required in the practical fabrication process of laddered pattern device, which may cause a decrease in yield and an increase in cost.

Metasurface has been reported with its powerful light manipulation capability in subwavelength scale [31], [32], [33], [34], which allows a full control of amplitude, phase, and polarization state for incident light. The introduction of a planarized meta-mirror array to replace the laddered patterned phase modulation layer has been reported to realize a planarized micro-OLED device with a subpixel size of 1.2 μm [35]. The planarized meta-mirror array only requires one step patterning and can simplify the following fabrication steps. The metasurface-driven optical cavity also provides a new technique route for micro-QLED patterning. However, sufficient nanorods are required for the realization of target function of designed meta-mirror, which limits the further minimization of subpixel size to submicron.

In this work, we proposed a novel metasurface-driven QLED (meta-QLED) structure with submicron subpixel size based on pixelated topological meta-mirrors. The proposed pixelated topological meta-mirror structure is introduced to construct meta-cavities with sufficient design freedom, which offers a stronger light manipulation capability for micron and submicron size meta-cavities with a limited period number. Based on the meta-cavity constructed by the optimized topological meta-mirrors, the pure color emission (energy ratio > 88 %) from RGB meta-QLEDs can be achieved. To prove the applicability of micro-QLEDs based on meta-cavities, micron and submicron full color meta-QLEDs are investigated. A relatively pure RGB color emission with target energy ratio larger than 85 % still can be remained with a subpixel size of 1 μm. And the minimum subpixel size can be further reduced to 0.6 μm with a 3 × 3 topological meta-mirror arrays. A full color meta-QLEDs device realized by topological meta-mirror array demonstrates one of the highest PPI among high resolution full color QLEDs. The proposed meta-QLED provides a new technique route for full color emission QLEDs, especially for the ultra-high pixel density required scenarios.

## 2 Results and discussion

### 2.1 Design and optimization of topological meta-mirror based meta-QLEDs

**Figure 1:**
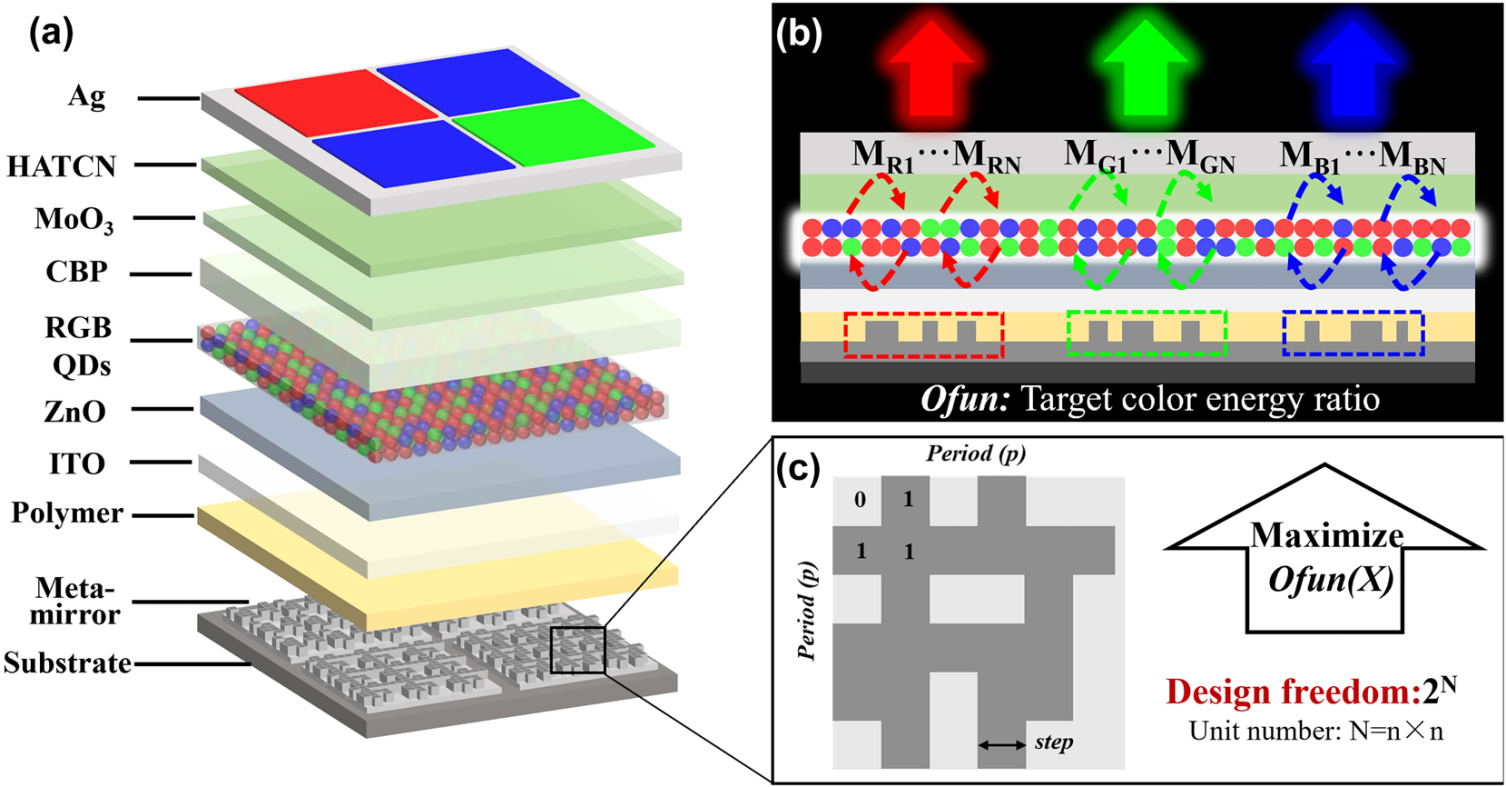
Device structure, target performance and emission mechanisms of full color meta-QLEDs. (a) Device structure of meta-QLEDs based on topological meta-mirror. (b) Target performance and emission mechanism of topological meta-cavities. (c) Topological meta-mirror structure and corresponding optimization parameters.

Here, the device structure for meta-QLEDs is shown in Figure 1(a). The device can be divided into two parts: the bottom topological meta-mirror layer with a planarization layer (polymer) and the top QLED device with a semi-transparent Ag as the anode, HATCN, MoO_3_, CBP layers as the functional hole injection layers (HILs) and hole transport layers (HTLs), a mixed RGB QDs layer as the emission layer, a ZnO layer as the electron transport layer (ETL), and a transparent ITO layer as the cathode. The Ag is chosen as the material for bottom meta-mirror because of its high reflectivity in visible bands as shown in Supplementary Figure 1, which ensures that more light will be reflected back and emitted at top surface. Meanwhile, compared with other metal material, the Ag-based meta-cavity demonstrates the best performance as shown in Supplementary Figure 12. And the possible oxidation problem of Ag in future applications can be avoided with suitable encapsulation technologies [36], [37], [38]. The meta-cavity is constructed by the bottom meta-mirror arrays and the semi-transparent (semi-reflective) top Ag electrode. As shown in Figure 1(b), the RGB color regions are defined by the bottom meta-mirror arrays with different structure parameters. For each color emission, the resonant cavity will be formed when the phase at target wavelength satisfies Equation (1) as followed [35], [39], [40]:

(1) $$\varphi_{\lambda ,\text{bottom}}+\varphi_{\lambda ,\text{top}}+4\pi \cdot \frac{n_{\lambda }\cdot L_{\text{cavity}}}{\lambda }=2m\pi$$

where *φ*_*λ*,bottom_ represents the bottom reflection phase, *φ*_*λ*,top_ represents the top reflection phase, *n*_*λ*_ ⋅ *L*_cavity_ represents the remained optical path in the QLED device, which is determined by each functional layer inside the QLED device as shown in Equation (2):

(2) $$\begin{array}{c} {\text{n}}_{\lambda ,\text{ITO}}\cdot L_{\text{ITO}}+{\text{n}}_{\lambda ,\text{ETL}}\cdot L_{\text{ETL}}+{\text{n}}_{\lambda ,\text{QDs}}\cdot L_{\text{QDs}}\\ \;+{\text{n}}_{\lambda ,\text{HTL}}\cdot L_{\text{HTL}}+{\text{n}}_{\lambda ,\text{HIL}}\cdot L_{\text{HIL}}={\text{n}}_{\lambda }\cdot L_{\text{cavity}} \end{array}$$

The top reflection phase *φ*_*λ*,top_ and the optical path of the cavity only change slightly due to the chromatic dispersion. To satisfy the resonant condition, the bottom reflection phase *φ*_*λ*,bottom_ should be precisely manipulated with the adjustment of bottom meta-mirror structure parameters. Here, the pixelated topological meta-mirror structures are utilized with an ultra-high design freedom as shown in Figure 1(c). Here, for a single meta-mirror period with *N* (*n* × *n*) unit cells, a total number of 2^*N*^ possible meta-mirror structures are provided for the realization of target performance. The exponentially growing optimization space provides sufficient design freedom for us. For example, the structure combination number can achieve 6.872 × 10^10^ with a fixed optimization step of 100 nm and a total pixel number of 36 in one unit cell. In addition, the optimization step can be further reduced to 50 nm, and the total pixel number can be increased or decreased to provide an even larger optimization parameter space. Compared with traditional nanorods-based meta-mirrors in meta-OLED [35], more design freedom is allowed for our proposed topological meta-mirror. For the same height of meta-mirror, only two structure parameters including the period and diameter can be adjusted in nanorods-based meta-mirror while there are *N* structure parameters (*n* × *n* cell) can be manipulated in our proposed topological meta-mirror. The increased structure possibility of pixelated meta-mirror provides more design freedom for meta-cavity devices, which also supports a stronger light manipulation capability with more complex structure, especially for ultra-small device size (submicron) with limited period numbers. To obtain the best structure parameters for target color emission, an inverse design method based on Bayesian optimization (BO) [41], [42] is utilized for bottom meta-mirror structure searching. During the optimization process, the energy ratio is used to evaluate the emission color purity, which can be defined by Equation (3):

(3) $$\text{Energy}\text{\_}\text{ratio}=\frac{E_{\text{target}}}{E_{\text{Red}}+E_{\text{Green}}+E_{\text{Blue}}}$$

where *E*_target_ represents the energy emitted in target color band region, *E*_Red_, *E*_Green_, and *E*_Blue_ represent the energy emitted in red, green, and blue color bands, respectively. Here, a high energy ratio represents a large amount of energy is emitted in the target color band, which indicates a good color purity. And the energy ratio is set as the target objective function (ofun) along with *N* meta-mirror structure parameters variables as shown in Equation (4):

(4) $$\text{Energy}\text{\_}\text{ratio}=\text{ofun}\left(x_{1},x_{2},x_{3}\ldots x_{N}\right)$$

where *x*_*N*_ represents the material setting of the *N* ^*th*^ unit cell in one period of topological meta-mirror. The value for each *x*_*N*_ is binary (0 represents for air and 1 represents for metal) as shown in Figure 1(c). The direct math expression between structure parameters and ofun is almost impossible to obtain due to the complex light–matter interaction in the meta-QLEDs. The ofun distribution inside the parameter space will be predicted by a surrogate function, which is constructed during the BO process by fitting the sample points in the parameter space. With the increase of optimization iterations, more points will be sampled. The difference between sampled point value and predicted value will be utilized to update the surrogate function and increase its accuracy. The optimization will end once reach a preset maximum iteration number. In the end of optimization, the global optimum point predicted by the surrogate function will be further verified to ensure a high energy ratio for target color emission. In this way, the best topological meta-mirror structures for pure RGB emission meta-QLEDs can be searched.

### 2.2 Optimized RGB meta-QLEDs by periodic topological meta-mirror

**Figure 2:**
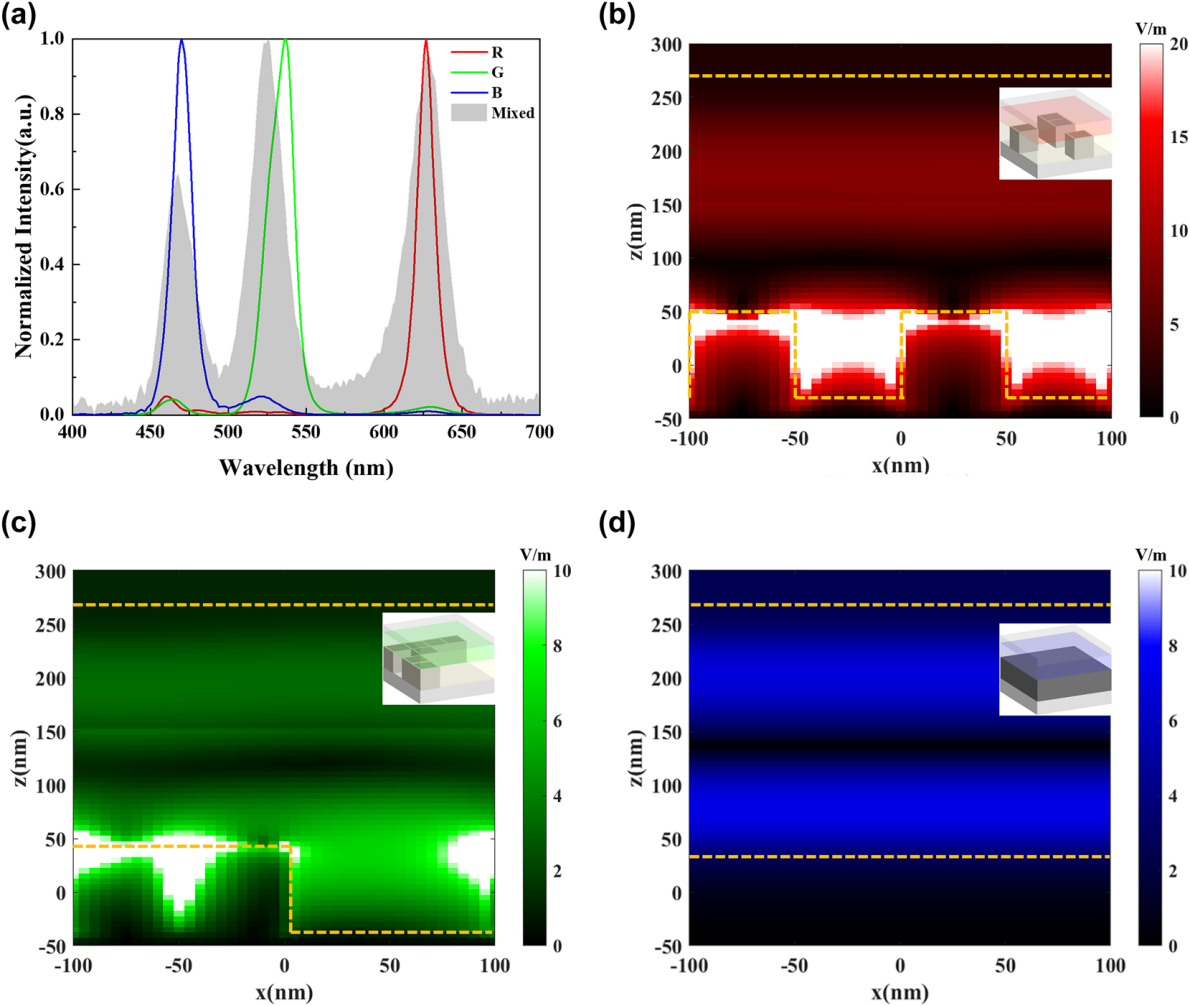
Optimized full color meta-QLEDs performance. (a) Emission spectrum of optimized RGB meta-QLEDs. Cross section (indicated by white dashed lines in the inset diagrams) electrical filed intensity distribution for (b) red, (c) green, and (b) blue meta-QLEDs at target resonance wavelengths (627 nm, 537 nm, and 470 nm). The orange dashed lines indicate the positions of top Ag reflector and bottom meta-mirror, and the insets 3D images demonstrate the material distribution inside one period (200 nm × 200 nm with 4 × 4 cells) of meta-QLED.

The optimization results for RGB meta-QLEDs based on topological meta-mirrors are shown in Figure 2 with an ideal periodic structure assumption, and the detailed spectrum performance is summarized in Table 1. To simplify the optimization and reduce the difficulties for meta-mirror fabrication, RGB meta-QLEDs are optimized based on a typical planar structure of microcavity QLED, which supports the basic resonance mode for blue light emission. In this way, only the optimizations for red and green meta-mirrors are required. For each color emission, the main emission peaks can be observed easily with a few unwanted peaks as shown in Figure 2(a). The gray area represents the emission of mixed RGB QDs emission in free space, which is a white light emission with relatively balanced RGB emission peaks. All the emission spectrums are normalized respectively for a better comparison. Here, due to the optimization target is to obtain pure RGB emission (high energy ratio), the emission peaks positions are allowed to have a slightly red or blue shift compared with original RGB emission. For the optimized green meta-QLEDs, the red shift of emission peaks allows a better color purity with a higher energy ratio. For all RGB meta-QLEDs, the energy ratios for the target color emission can be larger than 88 %, which indicates the pure RGB emissions from corresponding meta-cavity devices. Compared with original RGB emission of mixed QDs, the full width at half maximum (FWHM) for red, green, and blue emission modulated by meta-cavity can be reduced to 12 nm, 19 nm, and 15 nm with an original FWHM of 30 nm, 24 nm, and 22 nm, respectively. The narrower FWHMs prove the formulation of resonant cavities for each color emission. In our optimization process, the resonant cavity with stronger resonance mode intensity can be realized, but the suppress of unwanted light emission cannot be satisfied at the same time. As shown in Supplementary Figure 2, a stronger green resonance mode can be supported with a slightly changed meta-mirror structure, but the shift of target green resonance peak positions and increase of unwanted resonance mode peak intensity results in a decrease of target energy ratio to be less than 10 %. The intensity of target resonance mode along with the resonance mode in unwanted color emission should be considered both to realize a pure target color emission. With the construction of proper resonant cavities, the target color emission can be enhanced, the unwanted color emission is suppressed, and the pure RGB emission peaks in different meta-mirror regions can be realized.

**Table 1:** Device performance of the optimized RGB meta-QLEDs based on Ag topological meta-mirror array.

Color	Energy ratio	Peak wavelength	FWHM
Red	88.6 %	627 nm	12 nm
Green	90.1 %	537 nm	19 nm
Blue	88.4 %	470 nm	15 nm

To investigate the resonance mechanism for different meta-QLED, the cross-sectional electrical filed intensity distributions for RGB meta-QLEDs are simulated and plotted in Figure 2(b) and (c), the orange dashed lines demonstrate the two main interfaces inside the meta-QLED including the bottom Ag topological meta-mirror and the top Ag/Air interface, and functional layers including mixed QDs and charge transport layers are sandwiched between these two interfaces. The color bar represents the electrical filed intensity at different wavelengths (colors). The top view of one period of optimized meta-mirror structures for each color emission is demonstrated in the insets of Figure 2(b)–(d). Take consideration of fabrication limits, the minimum step is set as 50 nm with a height of 80 nm, which is corresponding with a low aspect ratio less than 2. And the period length is optimized as 200 nm, which allows more period numbers in a limited pixel size. The gray area represents the Ag, and the empty area will be filled up with planarized layer (polymer). Multiple resonance modes can be supported by these topological meta-cavities depending on the reflection phase adjustment of different meta-mirror structures, and the reflection phase difference will construct meta-cavities with different resonant wavelengths. The strongest electrical field intensity inside the device can be observed in the bottom topological mirror region due to the strong plasmonic resonance supported by the nano-sized metal structure. However, these strong resonance modes cannot be emitted outside of the meta-device because of the fast decay rate. The electrical field intensity in the functional layers should be concerned as it will directly influence the emitted light intensity. For RGB meta-QLEDs, strong electrical field intensity in the functional layers at different wavelength can be observed as shown in Figure 2(b)–(d), which indicates different resonance modes supported by different meta-cavities. Take the red meta-QLED as an example, the red light resonant meta-cavity is constructed while the blue light and green light are suppressed. The electrical filed intensity for red light demonstrates a much higher value compared with green and blue light as shown in Supplementary Figure 3(a), which indicates a much stronger resonant mode for red color emission (627 nm) compared with green and blue light. In this way, the red light emission can be enhanced in the red meta-QLED device while the blue and green light emission can be suppressed, which results in a strong red emission peak for red meta-QLED as shown in Figure 2(a). Similarly, the green light and blue light emission are enhanced in corresponding meta-QLED devices while the unwanted color emissions are suppressed as shown in Supplementary Figure 3(b) and (c). Meanwhile, compared with traditional QLED structure, the construction of target meta-cavity also gives an improvement of outcoupling efficiency as shown in Supplementary Figure 4, and the outcoupling efficiency of our meta-cavity is comparable with reported OLED meta-cavities [35].

### 2.3 Micron and submicron RGB meta-QLEDs by pixelated topological meta-mirror

**Figure 3:**
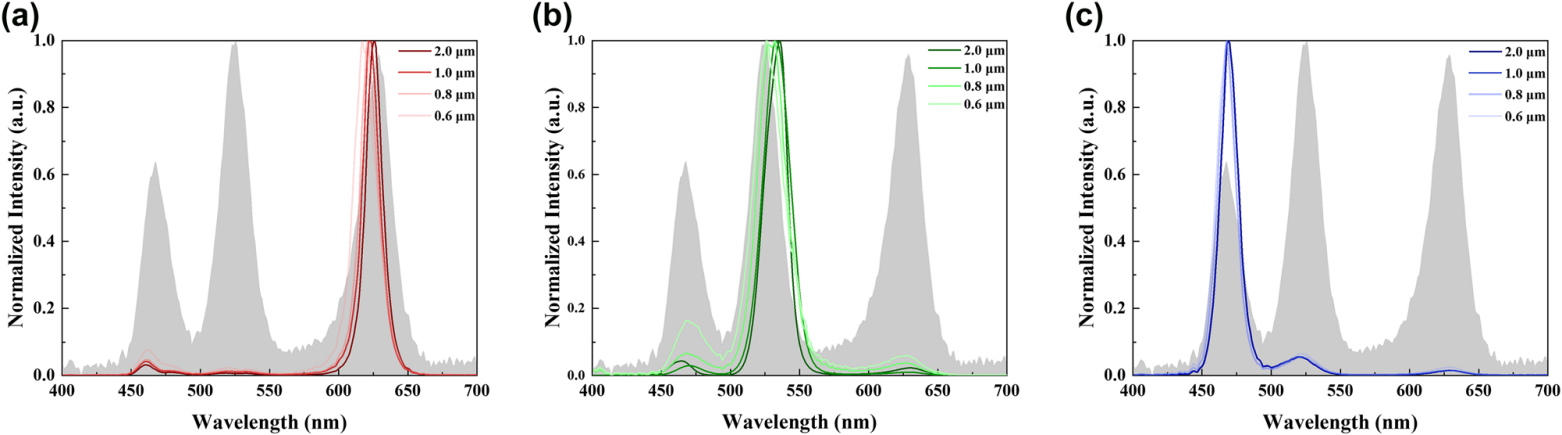
Emission spectrum of different sizes meta-QLEDs based on pixelated topological meta-mirror structure for (a) red light, (b) green light, and (c) blue light.

The meta-QLEDs can work well for pure RGB emission under the ideal periodic condition. However, in real display applications, infinite periods assumption for meta-mirror array is not practical due to the finite pixel size in a display screen. As a critical parameter for display, a smaller pixel size (larger pixel density) can improve the display resolution. Meanwhile, the ultrasmall size (micron or even submicron) subpixels are required in near eye display scenarios with a small display area. For meta-cavity based full color micro-QLEDs, the limited pixel size will reduce the number of periods. The topological meta-mirrors are designed based on ideal periodic assumption, which requires a sufficient period number to ensure the realization of target function. The limited number of periods may cause a deviation of target reflection phase manipulation, which will weaken resonance mode generated by the periodic structure and cause a performance degradation for meta-cavities with a decreased emission intensity of target color emission and an increased emission intensity of nontarget color emission. To investigate the effect of limited period number on meta-cavities performance, micron and submicron size meta-QLEDs are simulated and compared as shown in Figure 3. Here, the emission spectrums of RGB meta-QLEDs with different sizes from 2.0 μm to 0.6 μm are plotted in Figure 3(a)–(c). For the 2.0 μm meta-QLEDs, the FWHM narrowing effect still can be clearly observed as shown in Supplementary Table 1, which proves the construction of resonant cavities with similar intensity compared with ideal periodic situations. With the decreasing of device size, the resonant mode intensity will be reduced with shifting emission peaks, and the FWHM of each color emission will increase as well. With the decreasing period numbers, the realization of target functionality of meta-mirror arrays becomes a challenge. Benefits from the design concept of topological meta-mirrors, more complex material distributions (more supporting modes) inside one period are allowed even with a small period length compared with simple geometric structures such as nanocubes or nanorods. Here, the preset 200 nm period length allows a 10 × 10 topological meta-mirrors array for a 2 μm meta-QLED device, even for an ultrasmall subpixel size of 0.6 μm, a 3 × 3 topological meta-mirrors array is still supported. In this way, the target function for each topological meta-mirror can be realized, which ensures the color selective function for meta-cavities. Take the red meta-QLEDs as an example, the main red emission peak can be easily observed with different device size as shown in Figure 3(a). Only slightly blue shift occurs with the decreasing device size, and the relative intensities of unwanted emission peaks in green and blue region are increased slightly as well. Similar effect can be observed in Figure 3(b) and (c) for green and blue meta-QLEDs. These results further prove the color selective functionality of meta-cavities under micron and submicron conditions.

To quantitatively analyze the color purity degradation of RGB meta-QLEDs in micron and submicron scale, the energy ratios for pixelated topological meta-mirror based RGB meta-QLEDs are summarized as shown in Table 2. For a subpixel size of 2 μm and 1 μm, the target energy ratios for RGB meta-QLEDs are larger than 85 %, which is still very close to the ideal periodic assumption. The target color energy ratios are reduced with the decreasing device size. Higher energy ratios for RGB emission represent better color purities, which can cover a larger color gamut area. As shown in Supplementary Figure 5, the micron size RGB meta-QLEDs have a larger color gamut coverage compared with submicron RGB meta-QLEDs. The decreased energy ratios are mainly caused by the increased intensity unwanted emission peaks in the nontarget color region, which can be easily observed in Figure 3(a)–(c) especially for green light meta-QLEDs. Different from red and green meta-QLEDs, the blue light meta-QLEDs demonstrate a minimum color purity degradation based on their flat metal mirror structure. As a special situation for topological meta-mirrors (with full material occupation inside the target optimization space), the period length of blue meta-QLEDs do not equal to 200 nm but an arbitrary value. In this way, the period number can be much larger than red and green meta-QLEDs, which weaken the degradation of emission color purity effect. In the following parts, only the red and green meta-QLEDs will be discussed for their emission performance in micron and submicron scale for a fair comparison.

**Table 2:** Target color energy ratios summary for different size RGB meta-QLED devices based on pixelated topological meta-mirrors.

Color Size	2.0 μm	1.0 μm	0.8 μm	0.6 μm
Red	88.5 %	86.3 %	84.1 %	75.3 %
Green	89.8 %	89.6 %	79.7 %	69.3 %
Blue	87.2 %	87.1 %	82.4 %	80.6 %

**Figure 4:**
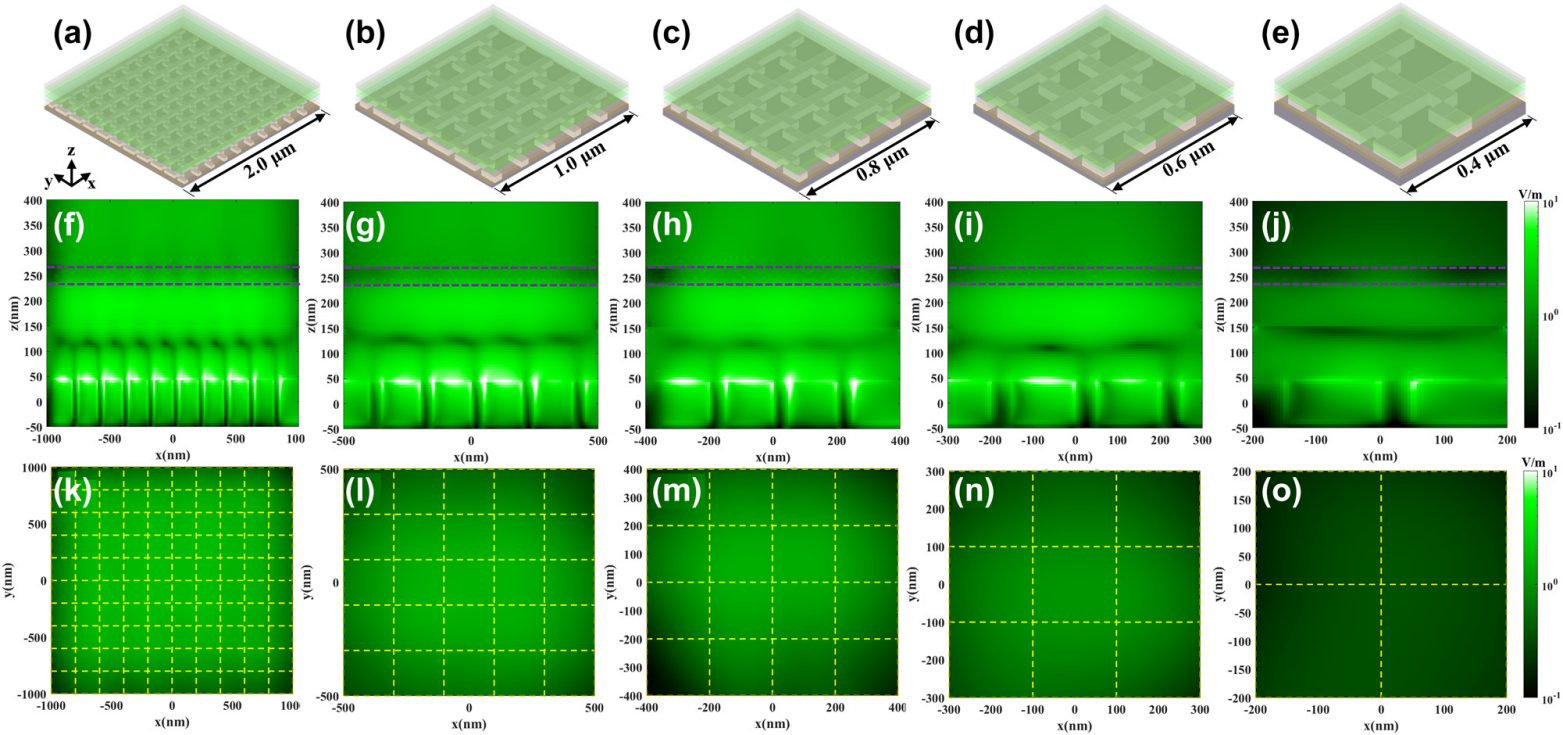
Schematic diagrams of green meta-QLED devices based on topological meta-mirror with a device size of (a) 2.0 μm (10 × 10 array), (b) 1.0 μm (5 × 5 array), (c) 0.8 μm (4 × 4 array), (d) 0.6 μm (3 × 3 array), and (e) 0.4 μm (3 × 3 array). The corresponding electrical field intensity distributions at cross section in device with a meta-QLED size of (f) 2.0 μm, (g) 1.0 μm, (h) 0.8 μm, (i) 0.6 μm, (j) 0.4 μm and at top view in air with a meta-QLED size of (k) 2.0 μm, (l) 1.0 μm, (m) 0.8 μm, (n) 0.6 μm, and (o) 0.4 μm. The purple dashed lines indicate the positions of top semi-transparent Ag electrode, and the yellow dashed lines indicate the positions of each period of meta-mirror inside the meta-QLED devices.

To further investigate the reason for the different color performance of topological meta-mirror based micron and submicron size meta-QLEDs, the electrical field intensity distribution inside and outside the green meta-QLED devices are plotted in Figure 4. As shown in the schematic diagrams in Figure 4(a)–(e), the total number of 10 × 10, 5 × 5, 4 × 4, 3 × 3, 2 × 2 meta-mirror periods are allowed for different subpixel sizes of 2 μm, 1 μm, 0.8 μm, 0.6 μm, and 0.4 μm. The cross-sectional monitors are set in the middle of device (*x*–*z* plane with *y* = 0) to record the electrical field intensity distributions inside the device. The top monitors are set in the *x*–*y* plane with a distance of 100 nm far from the top emitting surface of meta-QLEDs, which can be used to compare the emitting intensity in air without the interference of near-field outcoupled light such as evanescent light. With the decrease of device size, the green light resonance mode intensity decreases as well. The significant decline of green light resonance mode inside the device occurs between 0.6 μm and 0.4 μm as shown in Figure 4(f)–(j), and the emitted green light intensity in air also demonstrates a significant decay when the device size is reduced from 0.6 μm to 0.4 μm. The quantitatively top electrical filed intensity comparison for different sized green meta-QLED devices summarized in Supplementary Figure 6 further proves this observation result. For device sizes range from 2.0 μm to 0.6 μm, the green light intensity only decreases slightly both inside and outside the meta-QLED devices. Meanwhile, the unwanted resonance modes at red and blue light band do not demonstrate a significant increase as shown in Supplementary Figure 7. Even with a decreased subpixel size of 0.6 μm, the blue and red resonant modes are still much weaker than the green resonant mode, which ensure the main emission peaks at green band. With the decreased device size, fewer meta-mirror arrays are allowed, and the intensity of target resonant mode will be reduced, which causes a decreasing peak emission intensity as shown in Supplementary Figure 8(a) for green light meta-QLEDs. Notably, the green emission peak intensities drop dramatically when the device size is reduced from 0.6 μm to 0.4 μm, which is well-matched with electrical field distributions demonstrated in Figure 4 and Supplementary Figure 7. Although the emission peak intensities for red and blue light are reduced along with the green emission peaks as shown in Supplementary Figure 8(a), the decreasing speed for green light peak intensity is faster than unwanted red and blue light, which causes the increase of relative peak intensities for red and blue light shown in Figure 3(b), and the decrease of green light emission energy ratio summarized in Table 2. Similar decreasing peak intensities trend for red meta-QLED devices with different sizes can be observed in Supplementary Figure 8(b), and the dramatic peak intensity drop occurs between 0.6 μm and 0.4 μm as well. And the corresponding cross section electrical field distributions for red meta-QLEDs with different sizes are shown in Supplementary Figure 9. Compared with unwanted emission colors, the red light resonant modes dominate the device from 2.0 μm to 0.6 μm. The larger differences between red light electrical field intensity and unwanted green and blue light intensities contribute to purer red light emissions compared with green light emissions in green meta-QLEDs. From the emission performance of red and green meta-QLEDs, a 3 × 3 topological meta-mirror array (0.6 μm × 0.6 μm) is sufficient to support pure target color emissions from meta-cavities.

**Figure 5:**
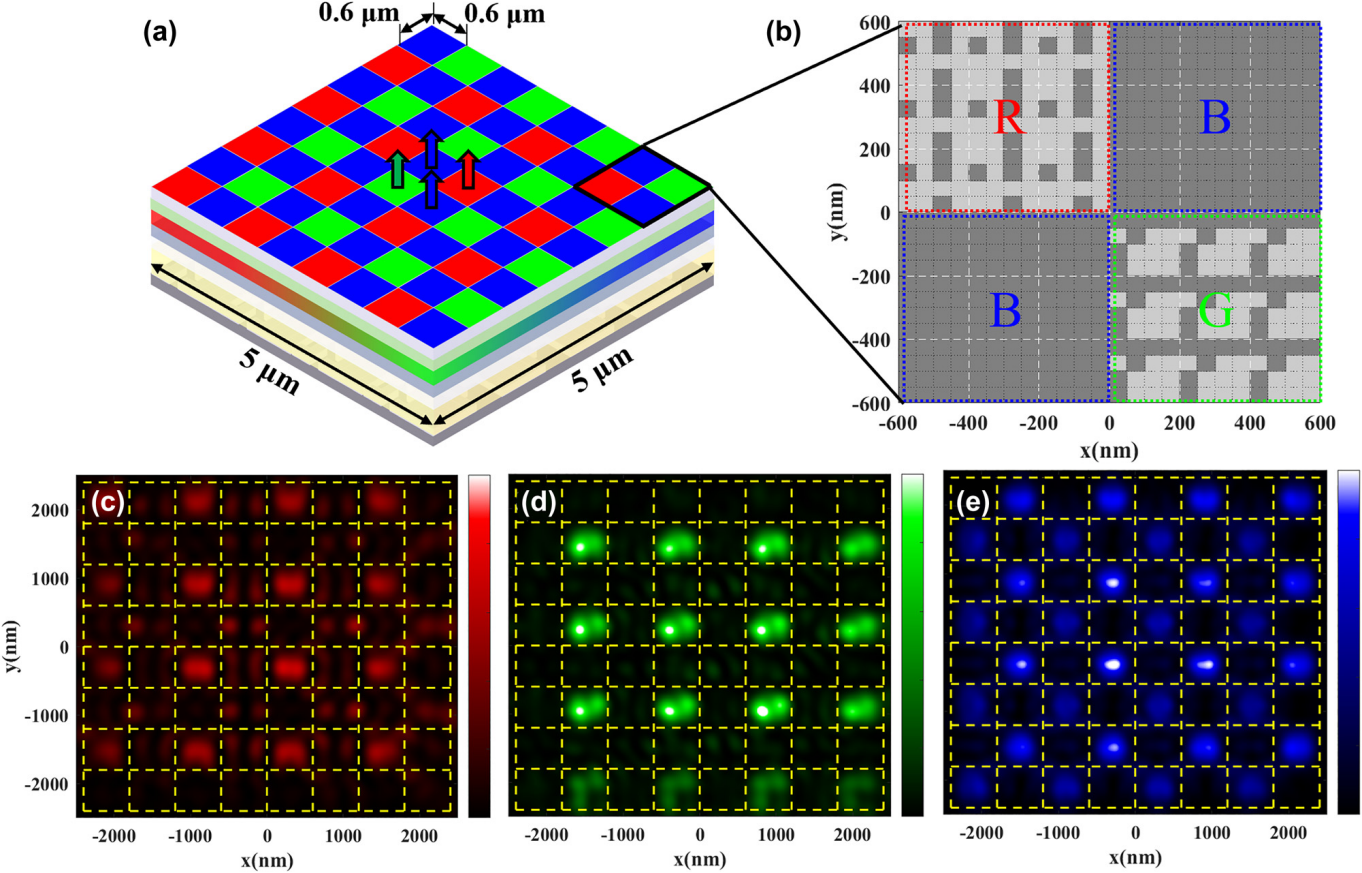
Meta-QLED devices with submicron RGB pixels. (a) Schematic diagram of a 5 μm full color meta-QLED device with a subpixel size of 0.6 μm based on pixelated topological meta-mirror. (b) Material distributions for RGBB pixelated topological meta-mirrors, the gray area represents the Ag. Top view of emitted light electrical field distribution of (c) red light, (d) green light, and (e) blue light. The yellow dashed lines represent the positions of subpixels.

To further prove the application potentials of pixelated topological meta-mirror based submicron meta-QLEDs, a 5 μm full-color QLED device is simulated with a subpixel size of 0.6 μm as shown in Figure 5(a). Different pixelated topological meta-mirror arrays are utilized for RGB emission areas as shown in Figure 5(b). The RGB dashed boxes indicate the positions of RGBB arranged subpixel region, and the white dashed boxes represent the positions of each period of meta-mirror in each color region. Here, a monitor is set at a distance of 100 nm far from the top emission surface of meta-QLED to record the emitted light intensity in air, which avoids the interference of nonsignificant near field light such as evanescent wave. The recorded electrical field intensity at different wavelengths (colors) is shown in Figure 5(c)–(e). The yellow dashed lines indicate the positions for each subpixel with the same arrangement as shown in Figure 5(a). For a more practical meta-QLED device with RGBB pixelated array, the crosstalk effect between adjoint subpixels should be considered as well, especially for small subpixel size with closer distance between the center of adjoint subpixels. Here, benefits from the strong light manipulation capability of topological meta-mirror even with limited period numbers, the strongest RGB emissions regions are perfectly matched with the target subpixels regions indicated by yellow dashed boxes for a subpixel size of 0.6 μm. For the 5 μm meta-QLED device, the situation of subpixels in the middle is more similar to ideal periodic situation, where each subpixel is surrounded by infinite subpixels. In this way, the resonant intensity of the subpixels in the middle can be higher with the support of adjacent subpixels, where the nontarget light emission mode may be outcoupled by different subpixel regions and become target light emission mode. On the other hand, fewer adjacent subpixels cause weaker emission intensities for the subpixels in the edge regions as shown in Figure 5(c)–(e). The RGBB arranged full color meta-QLED under ideal periodic assumption performance shown in Supplementary Figure 10 further prove this reason with same emission intensity for the same color subpixel in different regions. These results further prove the applicability of pixelated topological meta-mirror for an ultrahigh pixel density of 21,666 PPI full color meta-QLED device.

## 3 Conclusions

In this work, a novel meta-QLED device structure is proposed for high pixel density patterning of full color QLEDs with submicron subpixel size. The pixelated topological meta-mirrors with sufficient design freedom are utilized to construct meta-cavities for RGB emission, and the target color energy ratios can be larger than 88 %, which indicates good color purity. Meanwhile, the micron and submicron meta-QLEDs based on topological meta-mirror are investigated. Benefits from the larger design freedom, meta-mirror with smaller period length can be realized, which allows more periods in the same subpixel size with stronger light manipulation capability. The target color resonant modes dominate the emission behavior of meta-cavities with different meta-mirror array numbers from 10 × 10 to 3 × 3. For a typical small subpixel size of 1 μm (5 × 5 array), the pixelated topological meta-mirrors can support a strong RGB emission resonance mode, and the energy ratios for RGB emission are still larger than 85 %. And the main RGB emission peaks can be supported down to a minimum subpixel size of 0.6 μm (3 × 3 array). Meanwhile, the RGB emission patterns from an RGBB-arranged meta-QLED array with an ultrahigh PPI larger than 21,000 further prove the feasibility of the topological structure based meta-cavities. The pixelated topological meta-mirror based meta-cavities can also be applied for other light emitting devices including traditional LEDs, OLEDs, and PeLEDs and provides a novel full color patterning method to realize the ultrahigh pixel density devices for near eye display scenarios.

## 4 Materials and methods

### 4.1 Optimization and numerical simulations of meta-QLEDs

The inverse design method is conducted by MATLAB software and Lumerical FDTD software. The Bayesian optimization algorithm is based on the built-in optimization function in MATLAB, and the emission spectrum and electrical field distributions of meta-QLEDs are simulated by Lumerical FDTD software based on 3D models. A detailed meta-QLED device structure based on topological meta-mirror is provided in Supplementary 1 with layer thickness and refractive index distributions. For ideal periodic structured meta-QLED, the periodic boundary condition is applied in the horizontal plane and perfectly matched layer (PML) boundary condition is applied in the *z* axis. For finite sized meta-QLEDs, the PML boundary condition is applied for all directions. The TE and TM polarized light sources are set in the middle of QDs layer and are simulated separately and summed up together with the consideration of unpolarized emission from QDs. The emission spectrum is calculated by multiplying the far-field transmission spectrum of each meta-QLED device and the original RGB QDs emission spectrum. For a fair comparison between different meta-QLED devices, the cross section electrical field intensity distributions are collected with the same monitor in the middle of the device, and the top view of emitted electrical field intensity distributions is recorded by a monitor in air with a same 100 nm distance from the top of device.

## Supplementary Material

Supplementary Material Details
