# Supplementary material for: Submicron quantum dot light-emitting diodes enabled by pixelated topological meta-mirror
Source: Nanophotonics. 2025 Jan 8;14(2):241–51. doi: 10.1515/nanoph-2024-0543 (PMC11806506; doi:10.1515/nanoph-2024-0543)
Supplement: Supplementary file 1 — Supplementary Material Details [file j_nanoph-2024-0543_suppl_001.pdf]

Supplementary Document: Submicron Quantum Dot Light-Emitting Diodes Enabled by Pixelated Topological Meta-mirror

Taikang Ye,[1,2] Tian Dadi[1,3], Dan Wu[4,*], Xiao Wei Sun[1] and Kai Wang[1,*]

[1]State Key Laboratory of Optical Fiber and Cable Manufacture Technology, Institute of Nanoscience and Applications, and Department of Electronic and Electrical Engineering, Southern University of Science and Technology, Shenzhen 518055, China
[2]Department of Electrical and Computer Engineering, National University of Singapore, Singapore 117583, Singapore
[3]Pengcheng Laboratory, Shenzhen 518055, China
[4]College of New Materials and New Energies, Shenzhen Technology University, Shenzhen, 518118, China
**wudan@sztu.edu.cn, wangk@sustech.edu.cn*

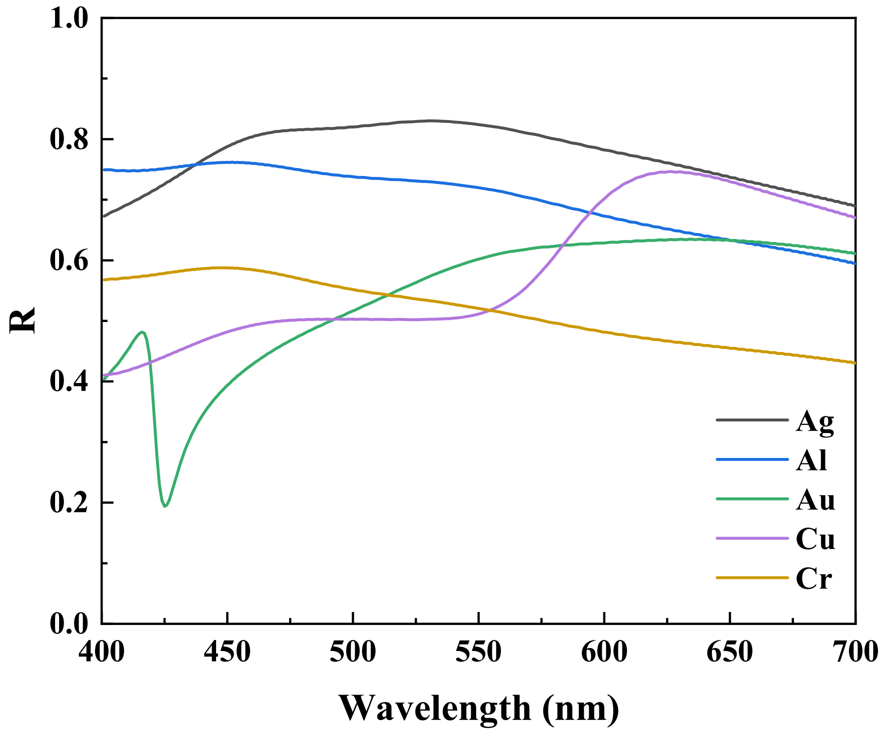

Supplementary Fig. 1 Simulated reflectivity of different metal materials in visible spectrum range.

Supplementary Table 1. Emission spectrum comparison between RGB meta-cavities (2μm size) and traditional RGB QLEDs.

Device type	Emission color	Peak wavelength	FWHM
Meta-cavities	Red	626 nm	14 nm
	Green	537 nm	19 nm
	Blue	469 nm	15 nm
Normal QLEDs	Red	629 nm	30 nm
	Green	526 nm	24 nm
	Blue	468 nm	22 nm

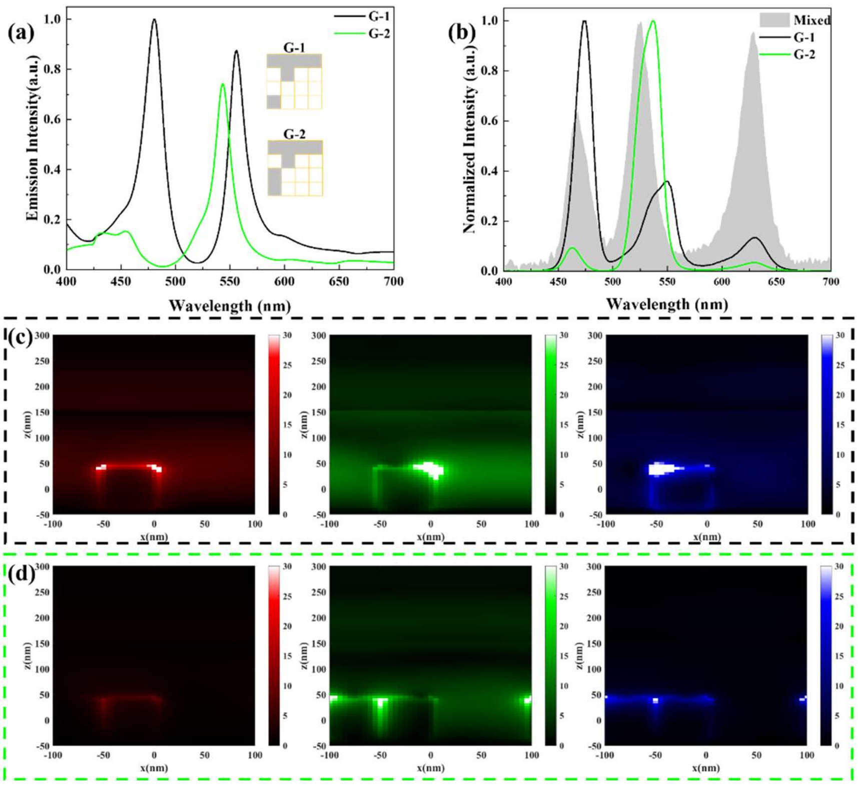

Supplementary Fig. 2. Normalized emission spectrum comparison between two different topological meta-mirrors based meta-QLEDs with light source of (a) white light with equal energy, (b) RGB mixed RGB QDs . Cross section electrical field intensity distribution of meta-QLED based on sample (c) G-1 and (d) G-2 at different RGB colors.

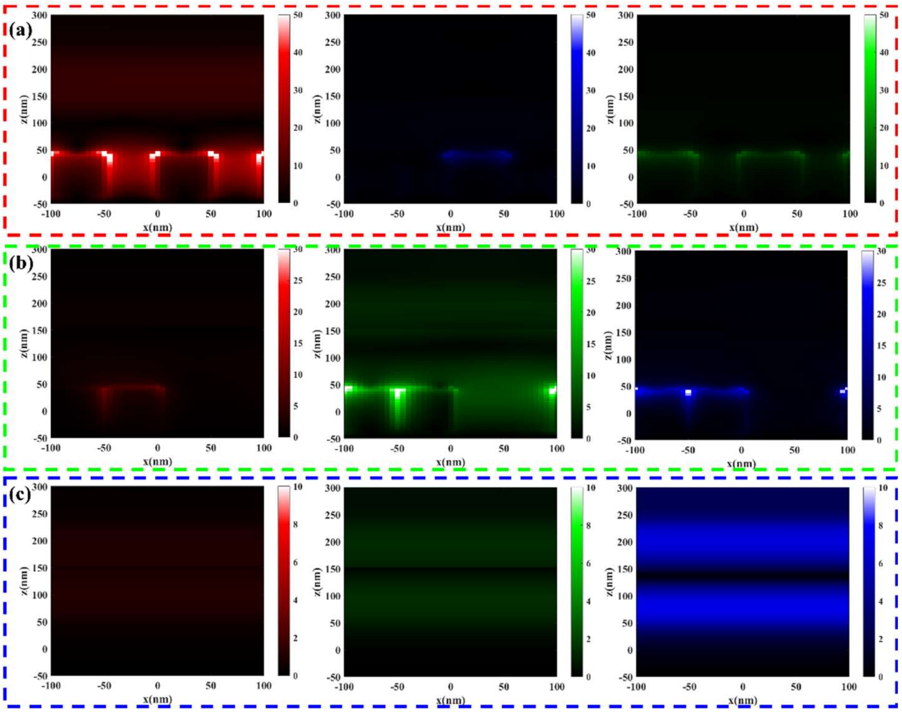

Supplementary Fig. 3 Device performance of optimized RGB meta-QLEDs based on meta-mirror. Electrical field intensity distribution for (a) red meta-cavity, (b) green meta-cavity, and (c) blue micro-cavity at different wavelengths(627 nm, 537 nm and 470 nm).

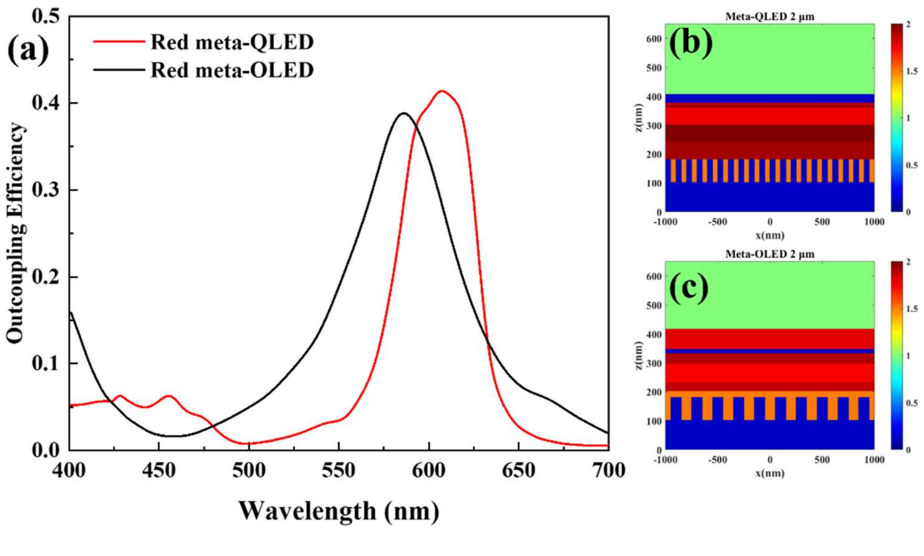

Supplementary Fig. 4. (a) Outcoupling efficiency comparison between meta-OLED and meta-QLED. Cross section material refractive index(n) distributions of simulated (b) meta-QLED and (c) meta-OLED. The blue areas represent the Ag.

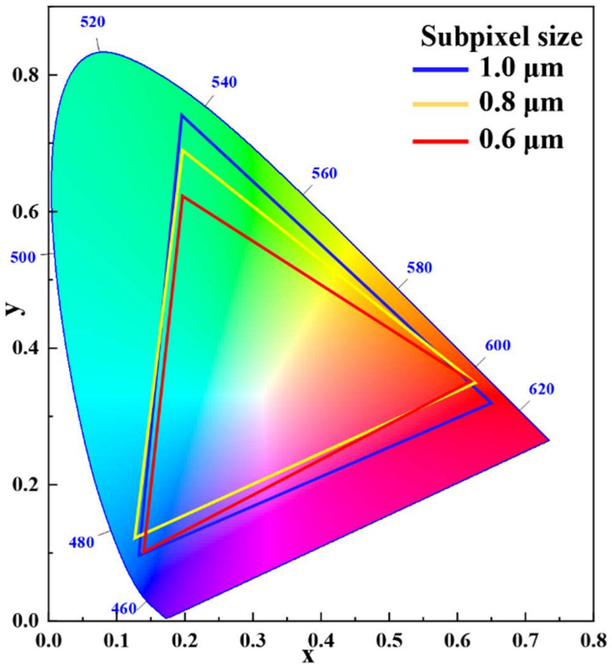

Supplementary Fig. 5 Color gamut coverage comparison between micron and submicron sized RGB meta-QLEDs.

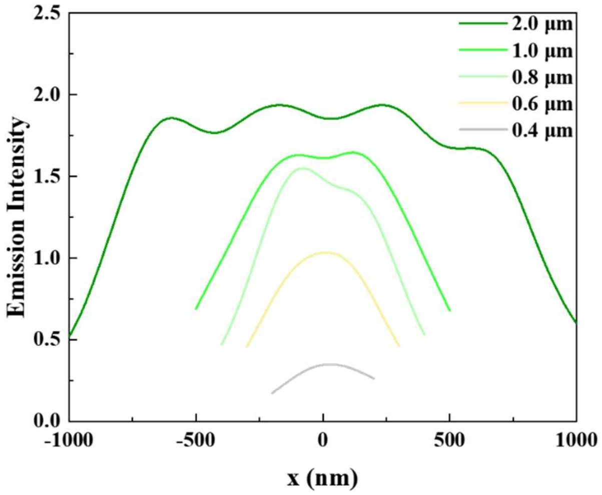

Supplementary Fig. 6. Emission intensity comparison for different size green meta-QLEDs.

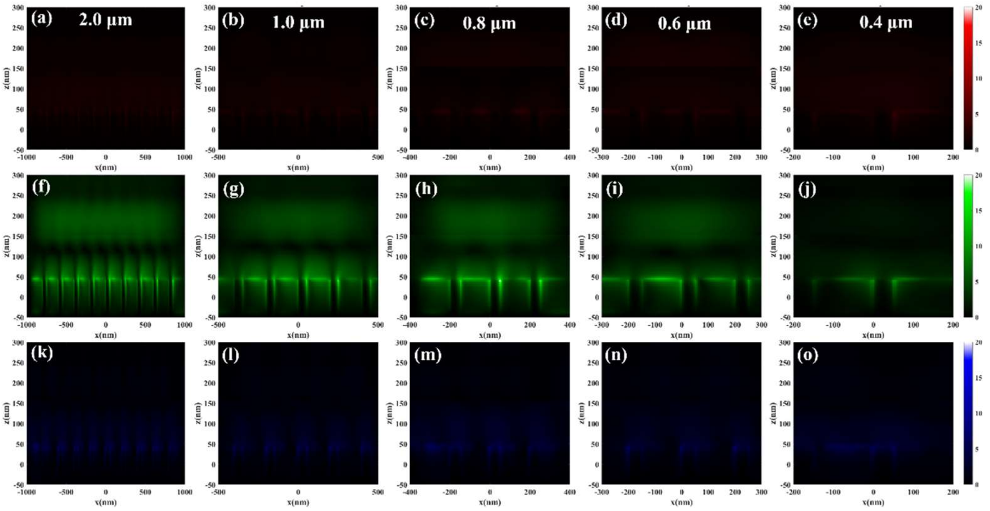

Supplementary Fig. 7 Cross section electrical field distributions of green meta-QLEDs. For red light (at 627 nm) with a device size of (a) 2.0 µm, (b) 1.0 µm, (c) 0.8 µm, (d) 0.6 µm, and (e) 0.4 µm. For green light (at 537 nm) with a device size of (f) 2.0 µm, (g) 1.0 µm, (h) 0.8 µm, (i) 0.6 µm, and (j) 0.4 µm. For blue light (at 470 nm) with a device size of (k) 2.0 µm, (l) 1.0 µm, (m) 0.8µm, (n) 0.6 µm, and (o) 0.4 µm.

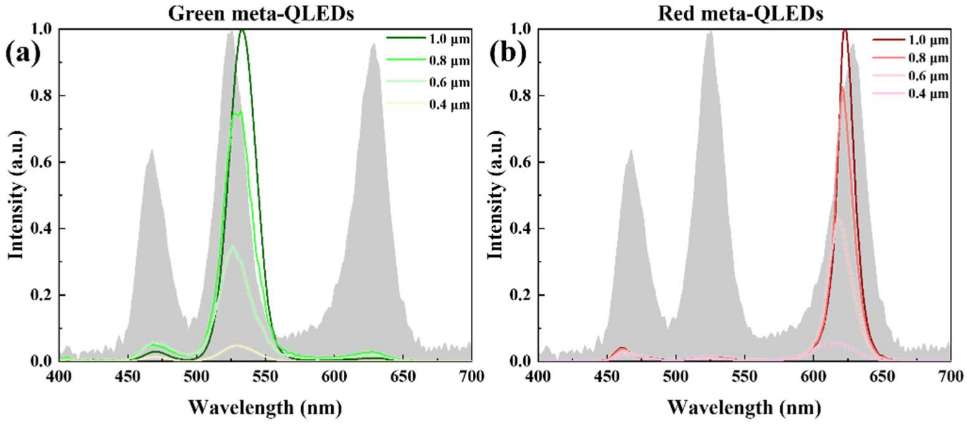

Supplementary Fig. 8 Fairfield emission intensity comparison between different size (1.0 µm to 0.4 µm) meta-QLEDs with optimized (a) green topological meta-mirror and (b) red topological meta-mirror.

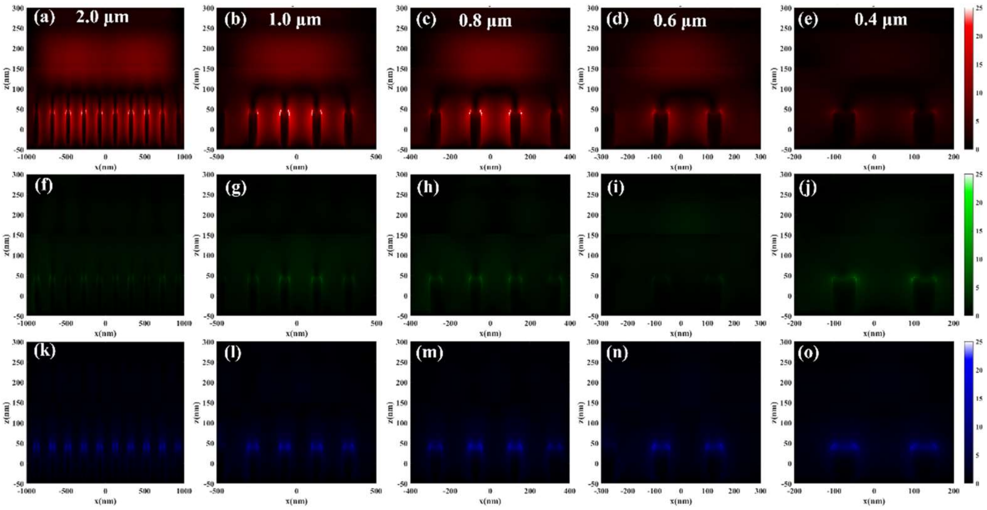

Supplementary Fig. 9 Cross section electrical field distributions of red meta-QLEDs. For red light (at 627 nm) with a device size of (a) 2.0 μm, (b) 1.0 μm, (c) 0.8 μm, (d) 0.6 μm, and (e) 0.4 μm. For green light (at 537 nm) with a device size of (f) 2.0 μm, (g) 1.0 μm, (h) 0.8 μm, (i) 0.6 μm, and (j) 0.4 μm. For blue light (at 470 nm) with a device size of (k) 2.0 μm, (l) 1.0 μm, (m) 0.8μm, (n) 0.6 μm, and (o) 0.4 μm.

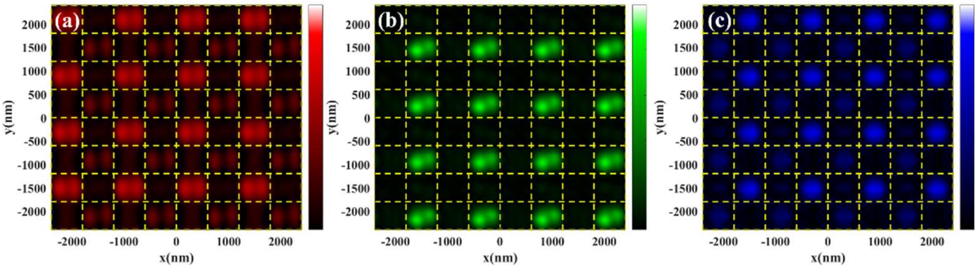

Supplementary Fig. 10 Full color meta-QLED device emission performance under ideal periodic situation. Top view of emitted light electrical field distribution of (c) red light, (d) green light, and (e) blue light. The yellow dashed lines represent the positions of subpixels.

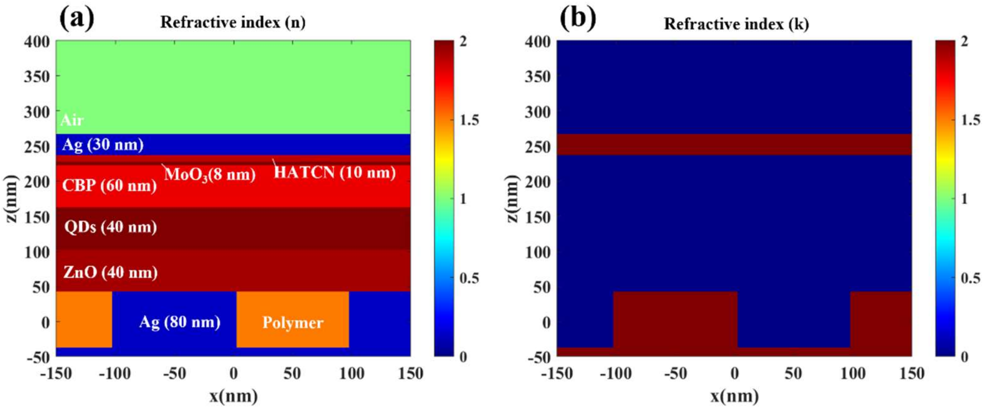

Supplementary Fig. 11 Cross section refractive index distributions of a meta-QLED based on topological meta-mirror. The thicknesses for different layers are indicated.

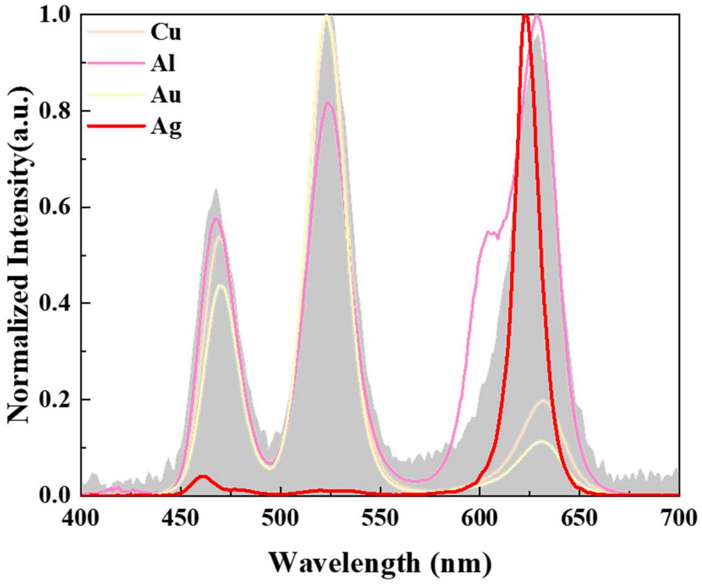

Supplementary Fig. 12 Emission performance of red meta-QLED based on different metal based optimized meta-mirror.
